# Inhibitory Effect of Kurarinone on Growth of Human Non-small Cell Lung Cancer: An Experimental Study Both *in Vitro* and *in Vivo* Studies

**DOI:** 10.3389/fphar.2018.00252

**Published:** 2018-03-23

**Authors:** Jie Yang, Hao Chen, Qiang Wang, Shihao Deng, Mi Huang, Xinhua Ma, Ping Song, Jingwen Du, Yun Huang, Yanzhang Wen, Yongshen Ren, Xinzhou Yang

**Affiliations:** ^1^School of Pharmaceutical Sciences, South-Central University for Nationalities, Wuhan, China; ^2^College of Pharmacy, Guangxi University of Chinese Medicine, Nanning, China; ^3^Division of Science & Technology, Qinghai University for Nationalities, Xining, China

**Keywords:** lung carcinoma, kurarinone, anticancer activity, apoptosis, multi-target

## Abstract

Kurarinone, a flavonoid isolated from *Sophora flavescens* Aiton, has been reported to have significant antitumor activity. However, the cytotoxic activity of kurarinone against non-small cell lung cancer (NSCLC) cells is still under explored. In our study, we have evaluated the inhibitory effects of kurarinone on the growth of NSCLC both *in vivo* and *in vitro* as well as the molecular mechanisms underlying kurarinone-induced A549 cell apoptosis. The results showed that kurarinone effectively inhibited the proliferation of A549 cells with little toxic effects on human bronchial epithelial cell line BEAS-2B. FASC examination and Hoechst 33258 staining assay showed that kurarinone dose-dependently provoked A549 cells apoptosis. Mechanistically, kurarinone significantly decreased the ratio of Bcl-2/Bax, thereby causing the activation of caspase 9 and caspase 3, and reduced the expression of Grp78, which led to relieve the inhibition of caspase-12 and caspase-7, as well as suppressing the activity of AKT. Meanwhile, modeling results from the Surflex-Dock program suggested that residue Ser473 of Akt is a potential binding site for kurarinone. *In vivo*, kurarinone inhibited the growth of A549 xenograft mouse models without apparent signs of toxicity. Our study indicated that kurarinone has the potential effects of anti-NSCLC, implemented through activating mitochondria apoptosis signaling pathway, as well as repressing the activity of endoplasmic reticulum pathway and AKT in A549 cells.

## Introduction

Lung carcinoma is the commonest type of cancer that contributes to increasing cancer death ratio globally (Mohan et al., 2016). NSCLC accounts for about 80–85% of lung carcinoma cases, with a comparatively low 5-year survival rate of <15% and merely 5–10% survival rate (Pore et al., 2013). Due to the limited curative effects and acute side effects, chemotherapy, radiotherapy, and immunotherapy in treating NSCLC remain not entirely as desired in spite of tremendous progress in lung carcinoma therapy (Niyazi et al., 2011). Therefore, more and more attention paid to anticancer agents derived from traditional Chinese medicines (TCMs), since their extensive efficacy and low toxicity (Abu-Surrah and Kettunen, 2006).

TCMs are experience-based remedies derived from 100 to 1000s of years of clinical applications, and have lots of advantages such as wide range of sources, low costs, and fewer side effects. Therefore, looking for biological active ingredients from TCMs to fight tumor has already become a new tendency for cancer treatment (Li et al., 2010). Very prominent examples for the success of TCMs include the DNA topoisomerase I inhibitor camptothecin from *Camptotheca acuminate* Decne. for use in non-Hodgkin’s lymphoma, acute lymphoblastic leukemia, and nephroblastoma in 1970s (Da Rocha et al., 2001), and the lignin podophyllotoxin isolated from *Podophyllum peltatum* L. in 1980s for the treatment of tumor (Canel et al., 2000), as well as ginsenoside Rg3 isolated from the roots of *Panax ginseng* C. A. Mey. which was discovered to treat lung, ovarian, breast, head and neck cancers in 2000s (Yang et al., 2012). It is not difficult to recognize that TCMs offer great potential for prevention and treatment of cancers.

According to Chinese pharmacy theories, as a TCM, *Sophora flavescens* Aiton can be applied in the therapy of fever, inflammatory disorders, acute dysentery, gastrointestinal hemorrhage, eczema, the treatment of malignant diseases, and so on. Particularly, kurarinone is abundant in *S. favescens* and has been demonstrated to have potent inhibitory effects on lung cancer both *in vivo* and *in vitro* (Sun et al., 2008). However, few articles have reported the cytotoxic activity of kurarinone against NSCLC cells and the molecular mechanisms underlying kurarinone-induced A549 cells apoptosis remained unclear.

As part of our continuing research in the discovering of new bioactive leads from TCMs as well as Chinese folk herbal plants (Wang et al., 2014; Yang et al., 2014, 2017), we undertook screening of a prefractionated TCM extract library. From the screening data, *Sophora flavescens* displayed strong cytotoxic activity. In the present study, we evaluated their cytotoxic activity and preliminarily elucidated the antitumor mechanism of kurarinone on A549 cell lines *in vivo* and *in vitro*.

## Materials and Methods

### General Experimental Procedures

UV and IR spectra were determined on a Shimadzu UV-250 spectrometer and a Shimadzu FTIR-8400S spectrometer, respectively. LC-PDA-ESIMS data were recorded on a Waters ACQUITY SQD MS system (Waters, Milford, MA, United States) connected to a Waters 1525 HPLC with a 2998 Photodiode Array Detector (Waters, Milford, MA, United States) and a Waters Sunfire^TM^C18 column (5 μm, 4.6 mm × 150 mm). NMR (MeOH-d4 or DMSO-d6) spectra were acquired on an AVANCE III 600 MHz NMR spectrometer equipped with Micro NMR tubes (1.4 mm). The chemical shifts (δ) were reported in ppm, and coupling constants (*J*) were given in Hz. The ESIMS and HRESIMS data were recorded on a Q-TOF Micro LC-MS-MS mass spectrometer. A Thermo C18 5 μm column (22 mm × 150 mm) was used for semi-preparative HPLC. A Waters 2535 HPLC fitted with a 2998 Photodiode Array Detector and a 2707 Autosampler was used for the semi-preparative separations. Silica gel (300–400 mesh, Yantai Jiangyou Silica Development, Co., Ltd., Yantai, China) were used for column chromatography. Silica gel GF254 precoated glass plates (1.00 mm, Yantai Jiangyou Silica Development, Co., Ltd., Yantai, China) were used for preparative TLC (PTLC).

### Plant Material

The roots of *Sophora flavescens* Aiton (family Leguminosae) were collected from Lingyuan City, Liaoning province, China in September, 2012, and identified by Professor Dingrong Wan of School of Pharmaceutical Sciences, South-Central University for Nationalities (SCUN), Wuhan, China. Avoucher specimen (No. SC0060) was deposited in School of Pharmaceutical Sciences, SCUN, Wuhan, China.

### Extraction and Isolation

Air-dried roots of *Sophora flavescens* Aiton (500 g) were triturated and then extracted sequentially by maceration with *n*-hexane (4 × 2.0 L, 5 h each) at room temperature, followed by ethyl acetate (4 × 2.0 L, 5 h each) and methanol (4 × 2.0 L, 5 h each). The solvents were evaporated at reduced pressure to yield 4.9, 36.8, and 58.7 g of *n*-hexane, ethyl acetate (SF-EtOAc), and methanol fractions, respectively.

The EtOAc extract (23 g) was subjected to a normal-phase silica gel column chromatography using a gradient solvent system of CH_2_Cl_2_-MeOH (1:0→50:1→30:1→20:1→10:1→5:1 →1:1→0:1, containing 0.1% formic acid) to give six major fractions Fr. A (1.5 g), Fr. B (3.3 g), Fr. C (4.2 g), Fr. D (3.9 g), Fr. E (3.1 g), and Fr. F (2.4 g). Fr. B was subject to a normal-phase silica gel column (EtOAc-Acetone, 100:1→30:1 →20:1→10:1→5:1→0:1, containing 0.1% formic acid) to afford four subfractions (B1–B4). 0.8 g of the B3 was further purified by PTLC followed by HPLC (H_2_O-CNCH_3_, 70%:30%→30%:70%, 40 min, containing 0.1% formic acid in both mobile phases) to give the purified compound and it has been elucidated as kurarinone by comparing its ^1^H, ^13^ C NMR and MS data (S1–S3 in Supplementary Material) with the reported literature (Kang et al., 2000).

### Cell Culture and Reagents

The NSCLC A549, NCI-H1975 and the human bronchial epithelial BEAS-2B cell lines were purchased from the American Type Culture Collection (ATCC; Manassas, VA, United States). The A549, NCI-H1975, and BEAS-2B cell lines were grown in a DMEM medium (Gibco) supplemented 10% fetal bovine serum (FBS) and 1% penicillin/streptomycin in a humidified atmosphere containing 5% CO_2_ at 37°C.

### MTT Assay

Cell viability of A549, NCI-H1975, and BEAS-2B were analyzed using 3-(4, 5-dimethylthiazol-2-yl)-2, 5-diphenyltetrazolium bromide (MTT) assay (Sai et al., 2015). Cisplatin (CDDP) was used as a positive control, since it is the most commonly prescribed cytotoxic agent for the treatment of NSCLC (Kang et al., 2016). Cells were seeded at 1 × 10^4^ cells/well into 96-well plates and were treated with kurarinone at the indicated dose of 200 μg/mL dissolved in DMSO. After 24 h, 100 uL of MTT solution was added to each well and incubated for 4 h. Then, DMSO was used to dissolve the formazan crystals and the absorbance was measured at 492 nm by a Microplate Reader (BIO-RAD). Then kurarinone was chosen for the further research owing to the good cytotoxicities. Subsequently, cells were incubated with kurarinone at 5, 10, 15, 20 μg/mL and CDDP at 25 μg/mL containing 1‰ DMSO for 12, 24, and 48 h, respectively. And then supernate was abandoned and 100 μL of MTT (5 mg/mL) was added to each well and incubated cells for 4 h. The formazan crystals were dissolved in DMSO and the absorbance was measured by a Microplate Reader (BIO-RAD).

### Observation of Morphological Changes

The A549 cells (1 × 10^5^/well) were seeded into 6-well plates, treated by kurarinone (0, 5, 10 μg/mL) and CDDP (25 μg/mL) for 24 h. Subsequently, a phase contrast microscope (Leica, Nussloch, Germany) was used to observe the cellular morphological changes.

### Hoechst 33258 Staining Assay

The A549 cells (1 × 10^5^/well) were seeded into 6-well plates and then treated with kurarinone (0, 5, 10 μg/mL) and CDDP (25 μg/mL) for 24 h. After discarding the supernatant, 1.0 mL of stationary liquid (methanol: acetic acid = 3:1) were covered for about 30 min. Then, Hoechst 33258 solution (5 μg/mL) was added to the wells for 30 min. And A549 cells were observed under a fluorescence microscope (Leica Microsystems, Wetzlar, Germany). Cells were scored apoptotic if the nuclei presented chromatin condensation, marginalization or nuclear beading (Mo et al., 2013).

### Flow Cytometry Analysis (FACS)

The A549 cells (1 × 10^5^ to 4 × 10^5^ cells per well) were seeded in 6-well plates, treated by kurarinone (0, 5, 10 μg/mL) and CDDP (25 μg/mL) for 24 h. And then cells were collected, fixed in 70% ethanol at 4°C overnight. Then the cells were washed in PBS and stained by 100 μL RNase A and 400 μL PI. The cell cycle distribution analysis was measured using a flow cytometer (BD Biosciences, United States) (Shen et al., 2014).

### Western Blot Analysis

Western blotting assay was used to analyze the expressions of apoptotic proteins in A549 cells *in vitro*. A549 cells were incubated with different doses of kurarinone for 24 h. Cells were lysed in lysis buffer, and incubated on ice for 30 min. After being centrifuged at 12,000 rpm for 15 min, the protein were separated by electrophoresis on 12% SDS-PAGE and transferred to polyvinylidine difluoride (PVDF) membrane (Bio-Rad). Membranes were blocked in 5% skimmed milk and incubated with primary antibodies Bax, Bcl-2, procaspase-9, procaspase-3, procaspase-7, procaspase-12, Grp78, Akt, Phospho-Akt (Ser473), β-actin, and GAPDH. The incubated mixture was washed with TBST. Then horseradish peroxidase (HRP) secondary antibodies were added with the mixture incubating at 37°C for 1 h. The incubated mixture was washed with TBST. HRP electrogenerated chemiluminescence (ECL) was used to develop, the developed films were taken and rinsed with pure water, and the washed films were dried. Scanning was used for recording.

### Molecular Docking

In order to explore the interaction and illustrate binding model for the active assay, molecular docking was carried out using Surflex-Dock of sybyl-x 1.10 program package (Tripos, St. Louis, MO, United States). The structural information from the theoretically modeled complex may help us to clarify the binding mechanism between Akt (PDB: 4GV1, this 3D crystal structure of Akt is replaced serine with aspartic acid in position 473 for simulation of Ser473 phosphorylation) and kurarinone (Addie et al., 2013). The 3D structure of kurarinone was also generated using SYBYL package. The standard bond lengths and bond angles, geometry optimization was carried out with the help of standard Tripos force field with a distance dependent-dielectric function, energy gradient of 0.001 kcal/mol and Gasteiger-Hückel as the electrostatics. The Ligand-Based Surface Calculation was employed to generate the protomol.

### *In Vivo* Xenograft Studies

Athymic nu/nu mice (BALB/c), 4–6 weeks of age, were purchased from the Beijing HFK Bioscience, Co., Ltd. (SCXK 2009-0015). Nude mice were implanted subcutaneously on the flank of the mice with 5 × 10^6^ A549 cells (0.1 mL/mouse). Once tumor size reached about 100 mm^3^, the mice were divided into four groups with 10 mice per group: kurarinone was administered i.p. at doses of 20 and 40 mg/kg/day. The CDDP group was administered i.p. at doses of 2.5 mg/kg/day every 2 days. The control group was injected with the same volume of PBS instead. The tumor volumes were calculated using the following formula: tumor volume (mm^3^) = 0.56 × length (mm) × width^2^ (square mm). After 27 days injection of kurarinone, mice were killed by cervical dislocation, and the subcutaneous tumors were harvested, weighed and fixed in 10% formalin for further examination.

### Statistical Analysis

All data were expressed as mean ± SD from three independent experiments. One-way analysis of variance (ANOVA) was used for multiple group comparisons by Tukey’s *post hoc* test using GraphPad Prism 5.0 software package. *P*-values < 0.05 were considered significant.

## Results

### Kurarinone Inhibits Proliferation of A549 Cells

**FIGURE 1:**
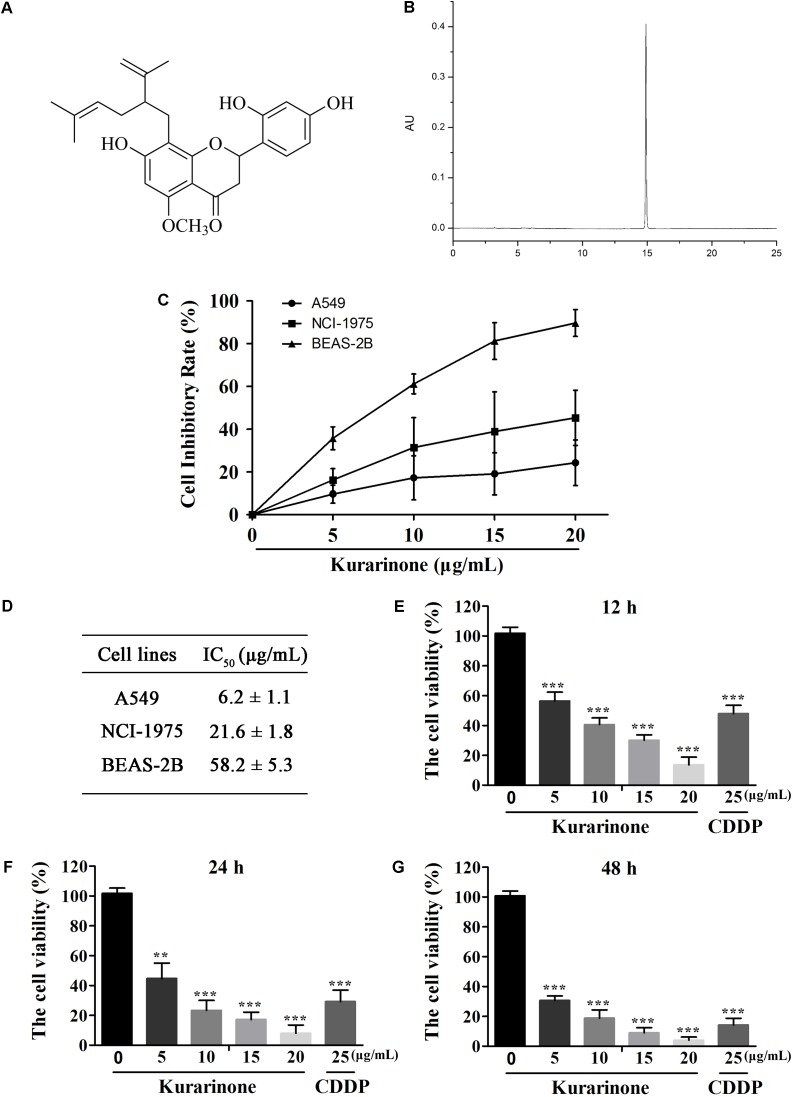
Kurarinone dose- and time-dependently provoked human A549 cell apoptosis *in vitro*. **(A)** Structure of kurarinone is shown. **(B)** HPLC chromatogram of kurarinone **(C)** A549, NCI-H1975 and the human bronchial epithelial BEAS-2B cells were incubated with 0, 5, 10, 15, 20 μg/mL of kurarinone for 24 h. **(D)** IC_50_ values of each cell line were calculated. **(E–G)** A549 cells were treated with indicated concentrations of kurarinone. After 12, 24, 48 h, cell viability was examined by MTT. The data are shown as the means ± SD of three independent experiments. ^∗^*P* < 0.05 compared to control, ^∗∗^*P* < 0.01 compared to control, ^∗∗∗^*P* < 0.001, compared to control.

Kurarinone (**Figures 1A,B**) was primarily tested for the percentages of growth inhibition on NSCLC A549 and NCI-H1975 cell lines using MTT assay. As presented in **Figure 1B**, kurarinone was potent to inhibit A549 cells proliferation with low toxic effects on the human bronchial epithelial cell line BEAS-2B (IC_50_ > 50 μg/mL). From the results, it can be deduced that kurarinone suppressed cell viability on A549 cells in time- and dose-dependent manners. In order to confirm this conjecture, the NSCLC cell line A549 was treated with kurarinone (0, 5, 10, 15, 20 μg/mL) and CDDP (25 μg/mL). After 12, 24 and 48 h of incubation, cell viability, and cytotoxicity were tested and shown in **Figures 1C–G**.

### Effects of Kurarinone on Apoptosis and Cell Cycle in A549 Cells

**FIGURE 2:**
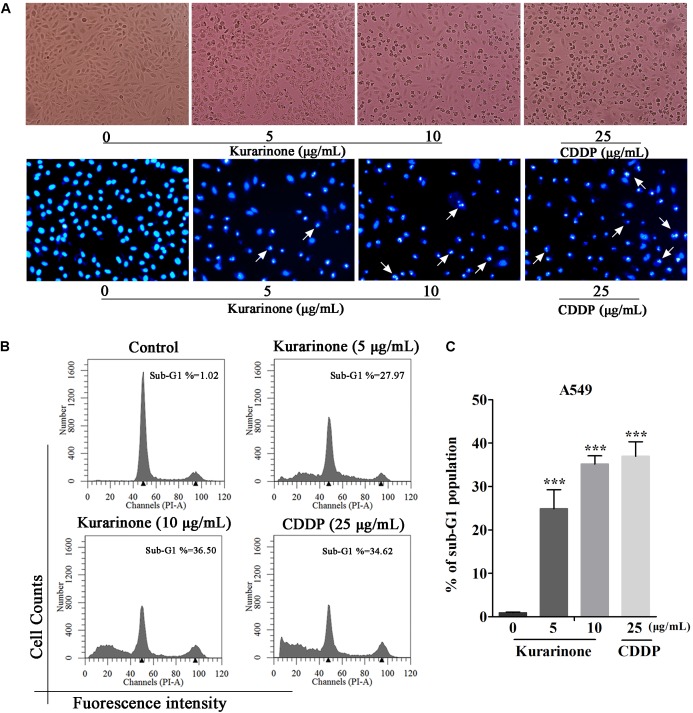
Kurarinone dose-dependently provoked A549 cell apoptosis *in vitro*. **(A)** A549 cells were treated with 0, 5, 10 μg/mL of kurarinone and 25 μg/mL of CDDP for 24 h, after which the cells were photographed under a phase contrast microscope. Then, the cells were fixed and incubated with Hoechst 33258 for 30 min, after which the cells were photographed under a fluorescence microscope. **(B)** Cell cycle distribution was observed by FACS. The result showed that kurarinone induced cell death in A549 cells in a dose-dependent manner. **(C)** The data are shown as the means ± SD of three independent experiments. ^∗^*P* < 0.05 compared to control, ^∗∗^*P* < 0.01 compared to control, ^∗∗∗^*P* < 0.001, compared to control.

After incubation for 24 h, we evaluate the influence of kurarinone on A549 cells such as distortion, membrane blebbing, and shrinkage by morphological observation with a phase contrast microscope. Results indicated that the shape of a majority of cells progressively showed shrinkage and necrosis (**Figure 2A**). Then the cells were identified by Hoechst 33258 staining and cells treated with kurarinone showed morphological changes characteristic of apoptosis, including chromatin condensation and nuclear DNA fragmentation (**Figure 2A**).

To explore the capability of kurarinone in triggering NSCLC cell apoptosis, A549 cells were treated with 5 and 10 μg/mL of kurarinone for 24 h. Then, the ratios of sub-G1 DNA in the cell population were determined by FACS. The results shown that A549 cells treated with kurarinone for 24 h dose-dependently promoted the percentages of sub-G1 DNA (**Figure 2B**). These data indicate that kurarinone is able to break cell cycle progression and prevent the cells from potentially becoming cancerous.

### Effects of Different Doses of Kurarinone on the Apoptosis of A549 Cell

**FIGURE 3:**
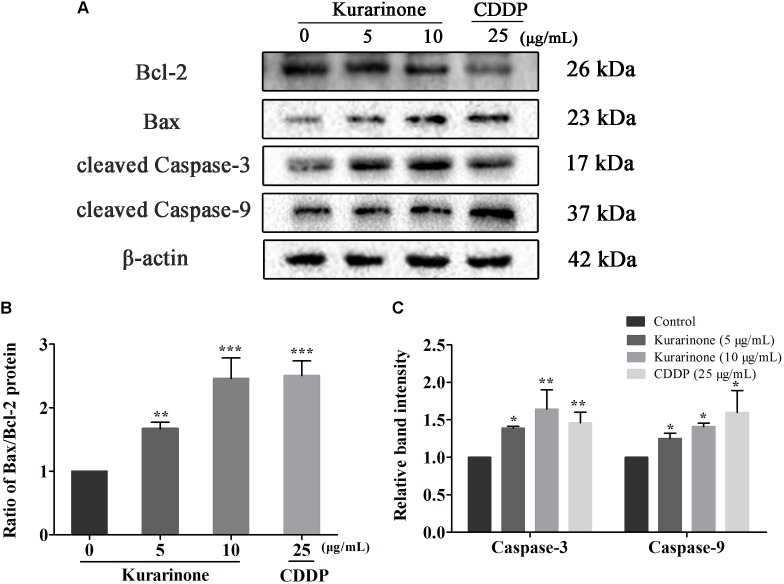
Effects of kurarinone on the apoptosis regulatory proteins in A549 cells. Bcl-2, Bax, caspase-3, and caspase-9 were assessed by western blot analysis. β-Actin was used as an internal control. **(A)** In A549 cells, expression of interacting protein was detected; **(B,C)** All data were shown as the means ± SD of three independent experiments. ^∗^*P* < 0.05 compared to control, ^∗∗^*P* < 0.01 compared to control, ^∗∗∗^*P* < 0.001, compared to control.

In the course of apoptosis, cyt C released from mitochondria to cytoplasm to down-regulate Bcl-2 expression and up-regulate Bax expression. During the apoptotic signaling network, caspases also play an important part, and apoptotic pathways relied on activation of caspases for the final execution of apoptosis (Zhao et al., 2016). Hence, we used western blotting analysis to verify the effect of apoptosis-related proteins by kurarinone (S4 in Supplementary Material). We found that kurarinone can up-regulated pro-apoptosis proteins cleaved-caspase-3, cleaved-caspase-9, Bax, and down-regulated antiapoptosis protein Bcl-2 expression (**Figures 3A–C**). It demonstrated that a mitochondria-dependent pathway is involved in kurarinone-induced apoptosis in A549 cells.

### Effects of Different Doses of Kurarinone on the Expression of ERS-Related Proteins and PI3K/AKT Signaling Pathway-Related Proteins in A549 Cells

**FIGURE 4:**
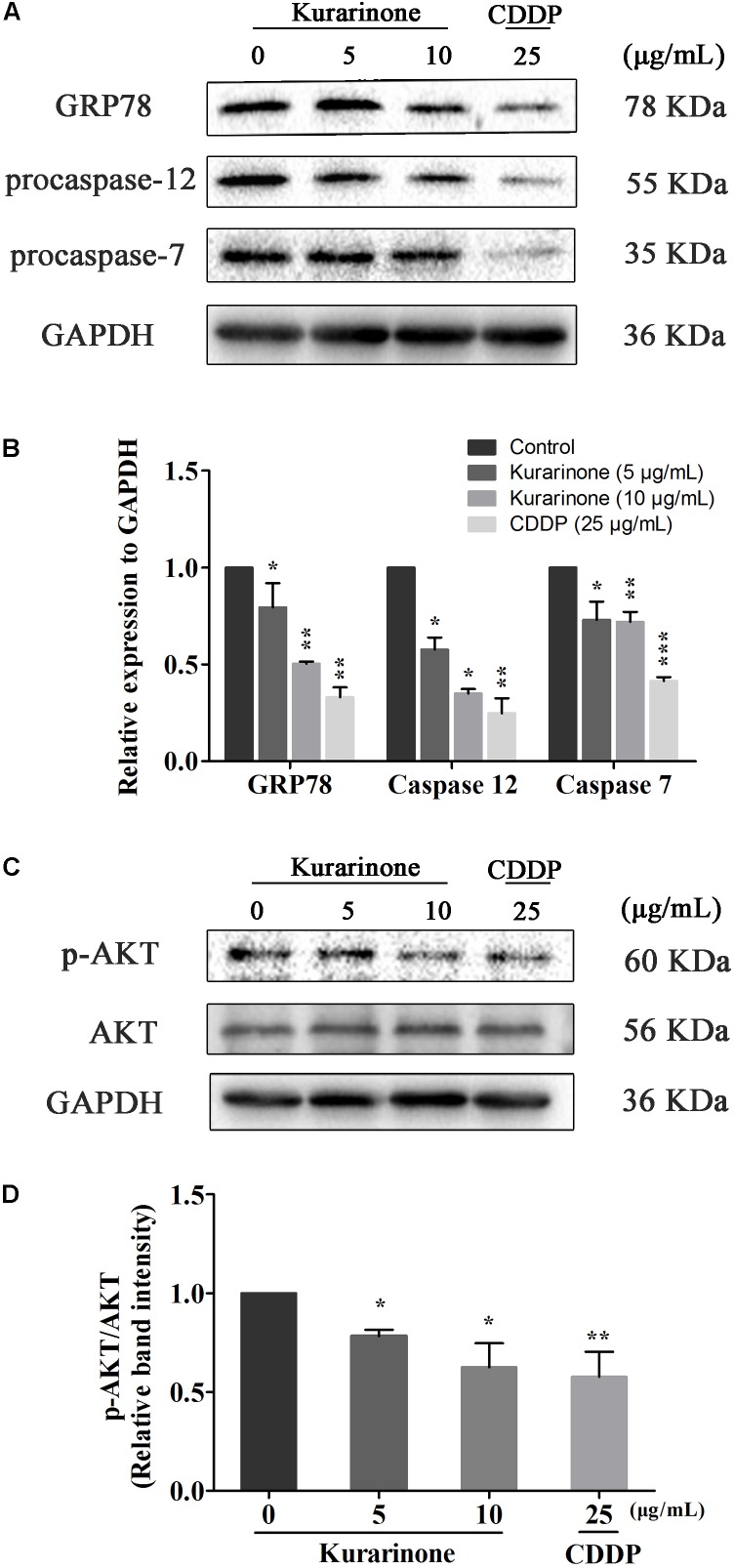
Kurarinone induced the expression of ER stress-related molecules and AKT in A549 cells. **(A,B)** The expressions of GRP78, procaspase-12, procaspase-7, and GAPDH were analyzed by Western blotting; **(C,D)** Akt activation is involved in the apoptosis. Kurarinone down-regulated the phosphorylation of Akt in a dose-dependent manner. All data are shown as the means ± SD of three independent experiments. ^∗^*P* < 0.05 compared to control, ^∗∗^*P* < 0.01 compared to control, ^∗∗∗^*P* < 0.001, compared to control.

Recent studies have also pointed out that the signaling pathway of apoptosis mediated by ERS was a new mechanism of apoptosis and ectopically expressed GRP78, caspase-7, and caspase-12 can form a complex to couple ER stress to the cell death program. In our study, we tested the protein levels of Grp78, procaspase-7, and procaspase-12 via western blot analysis (S5 in Supplementary Material). The results indicated that kurarinone down-regulated the expression of Grp78, procaspase-7, and procaspase-12 in a dose-dependent manner (**Figures 4A,B**).

AKT protein as a vital component of PI3K-AKT pathway is another important regulator of cellular proliferation and survival, and its dysregulation is associated with the development of cancer. Phosphorylation on Ser473 is essential for activation of AKT. In our study, we measured its phosphorylation level via western blot (S5–S6 in Supplementary Material). Kurarinone dose-dependently repressed the phosphorylation of AKT on Ser473 but did not affect protein levels of AKT (**Figure 4C**).

### Akt Ser473 Is a Potential Target for Regulating PI3K/AKT Signaling Pathway-Related Proteins in A549 Cells

**FIGURE 5:**
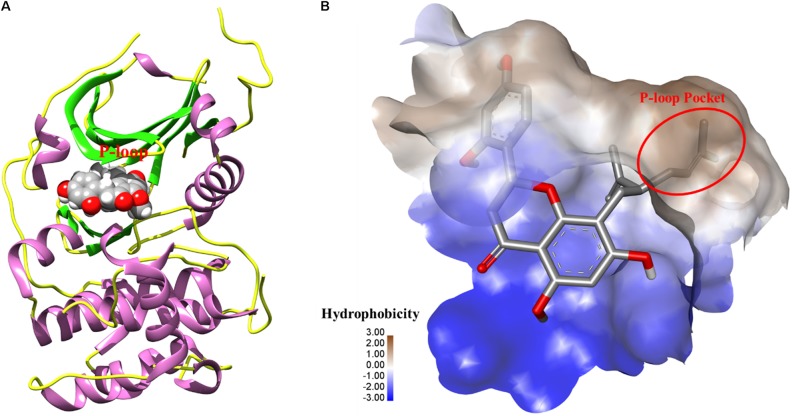
**(A)** The 3D-interaction was plotted by UCSF Chimera. The protein was showed as rounded ribbon. Kurarinone was showed as CPK model. **(B)** The binding pocket was rendered hydrophobicity properties by Discovery Studio Visualizer 4.1, from blue for hydrophilic to brown for hydrophobic.

To further elucidate the potential target for kurarinone, the interaction between kurarinone and Akt was analyzed by Surflex-Dock. The results indicated that the parameter set for the Surflex-Dock simulation was reasonable. From the docking results, we found that kurarinone bound with the kinase hinge region and the lavandulyl was very important to fill the P-loop hydrophobic pocket of AKT (**Figure 5**). It demonstrated that p-AKT might be the potential antitumor target of kurarinone.

### Kurarinone Inhibited the *in Vivo* Tumor Growth

**FIGURE 6:**
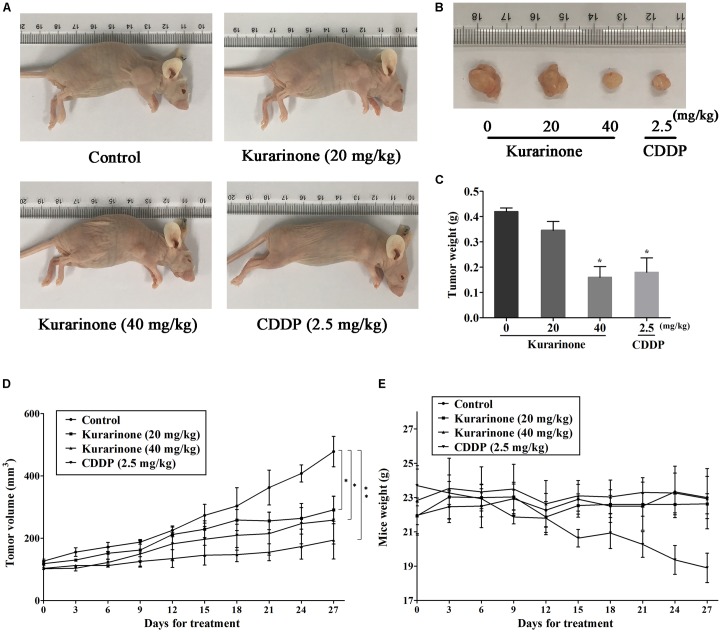
Kurarinone inhibited A549 xenograft growth in nude mice. Forty female BALB/c nude mice received an injection of A549 cells and were divided into four groups. Kurarinone and CDDP were administered at a dose of 20, 40 mg/kg/day and 2.5 mg/kg every 2 days. On day 27, mice were sacrificed and tumor xenografts were excised completely from tissues. **(A,B)** The representatives of the control and kurarinone-treated mice and tumors. **(C–E)** Statistical analyses demonstrated the tumor weights, tumor volume, and body weight. The data are shown as the means ± SD of three independent experiments. ^∗^*P* < 0.05 compared to control, ^∗∗^*P* < 0.01 compared to control, ^∗∗∗^*P* < 0.001, compared to control.

**FIGURE 7:**
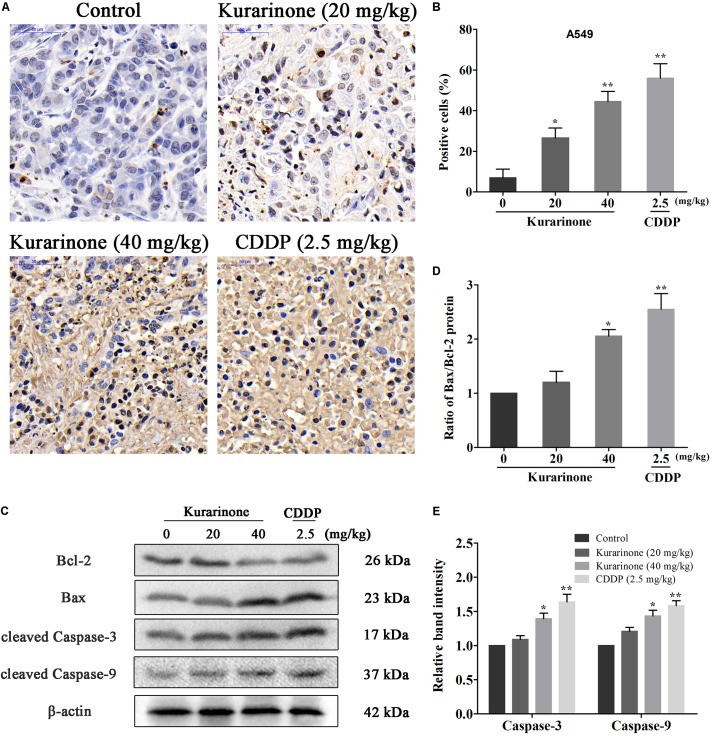
Kurarinone inhibited the *in vivo* tumor growth through activating the intrinsic apoptosis pathway. **(A,B)** Nude mice bearing A549 xenograft were sacrificed after treatment with kurarinone (20 and 40 mg/kg/day), and the tumors were harvested for TUNEL assay. The brown stains in Tunel assay in kurarinone treated tumor tissue suggested that kurarinone induced apoptosis in tumor of treated mice. **(C–E)** The tumors dissected from the nude mice were homogenated and subjected to the total protein extraction followed by western blot analysis to detect the expressions of Bcl-2, Bax, Bax, and the cleaved-caspases. All data are shown as the means ± SD of three independent experiments. ^∗^*P* < 0.05 compared to control, ^∗∗^*P* < 0.01 compared to control, ^∗∗∗^*P* < 0.001, compared to control.

The *in vivo* anticancer effect of kurarinone was assessed in tumor xenografted mouse model. A549 cells were injected on the frank of the mice subcutaneously to reach about the size of 100 mm^3^, and then the mice were assigned to four groups randomly: control group with PBS, kurarinone treated group (20 and 40 mg/kg/day), and CDDP treated group (2.5 mg/kg, i.p. every 2 days). On the last day of kurarinone treatment, the tumor volumes significantly reduced compared with the control group (**Figures 6A,B,D**). Compared with control, kurarinone at the concentrations of 20 and 40 mg/kg significantly decreased the mean tumor weight (*P* < 0.05 and *P* < 0.01) (**Figure 6C**). Kurarinone showed no detectable toxicity in all the groups since there were no statistically significant effects on body weight (**Figure 6E**), behavior, and appearance between the kurarinone treated groups and control group. To investigate whether kurarinone-induced apoptosis also occurred *in vivo*, tunel assay was performed to determine apoptosis in tumor graft in mice. As indicated by brown spots, cells underwent apoptosis after kurarinone treatment (**Figures 7A,B**). To further confirm our results, we detected the expression of related proteins in tumor tissues. The results showed that kurarinone can up-regulate pro-apoptosis proteins cleaved-caspase-3, cleaved-caspase-9, Bax, and down-regulate antiapoptosis Bcl-2 expression (**Figures 7C–E**).

## Discussion

In our present work, kurarinone exhibited distinct cytotoxic activity on A549 cells. Moreover, our results indicated that kurarinone resulted in cell morphological changes and reduced cell viability (**Figure 1**). Besides, at high doses, kurarinone strongly increased accumulation of sub-G1 phase, which illustrates that kurarinone strongly induce apoptosis of A549 cells (**Figure 2**). Collectively, these data suggested that kurarinone is capable of inducing A549 cells apoptosis.

The regulation of cell apoptosis may take effects through targeting specific proteins in different signal pathways, such as ER stress, mitochondrial dysfunction pathway, PI3K/AKT pathway, Fas/FasL pathway, MAPK signal pathway, and so on. In our study, western blot analysis was conducted to clarify the possible anticancer molecular mechanism of kurarinone. As previous publications reported, high ratio of Bax to Bcl-2 can result in the release of cyt c from the intermembrane space of mitochondria to the cytosol (Tophkhane et al., 2007). Then, the released cyt c produces the formation of apoptosome-containing apoptotic protease activating factor 1 (Apaf-1) and caspase-9 to activates the effector procaspases, including procaspase-3, to accomplish the process of apoptosis (Hill et al., 2004). In our study, kurarinone could down-regulate Bcl-2 and up-regulate Bax expressions, and the significant increases in caspase-3 and caspase-9 were also observed in kurarinone treated A549 cells. These results demonstrate that a mitochondria-dependent pathway is involved in kurarinone induced apoptosis in A549 cells.

Furthermore, kurarinone could also influence related proteins in ER stress. Earlier studies have demonstrated that the association of caspase-7 and caspase-12 with the ER compartment at the ER surface prevents their activation and release (Nakagawa and Yuan, 2000). Ectopically expressed GRP78, caspase-7, and caspase-12 can form a complex to inhibit the cell death program mediated by the activation of caspase-12 (Reddy et al., 2003). In our study, treatment of A549 cells with kurarinone resulted in a decrease in the ERS-related proteins (Grp78, procaspase-7, procaspase-12). It turned out that as a marker for pro-survival mechanism, the lower expression of GRP78 promoted the kurarinone-induced apoptosis in A549 cells. Meanwhile, ER stress-mediated activation of caspase-7 and caspase-12 illustrated that translocation of cytoplasmic caspase-7 to the surface prolonged ER stress facilitates movement of active caspase-12 into the cytoplasm (Rao et al., 2001) and induces cell death.

Normally, the mechanism of apoptosis is also limited by antiapoptotic pathways. Differed from the caspase family, AKT is well-known as the key molecular constituting significant antiapoptotic pathways in many cancers (Li et al., 2016). To preliminarily explore whether AKT signaling was the possible mediator of A549 cell apoptosis triggered by kurarinone, the activity of AKT were detected. The results confirmed our conjecture that kurarinone treatment inhibited the activity of AKT in A549 cells (**Figure 4**), suggesting that kurarinone-induced A549 cell apoptosis might be triggered by the inhibition of AKT. To understand the mechanism of how kurarinone produced inhibitory effect in A549 cells, we firstly utilized molecular docking to search for potential antitumor target of kurarinone. Modeling results suggested that p-AKT (Ser 473) might be the target of kurarinone, which was confirmed by the experiments that kurarinone decreased the expression of p-AKT in Western blot.

To further examine the antitumor effects and mechanisms of kurarinone, the *in vivo* experiments were performed in xenograft animal model. After the treatment of kurarinone, the growth of xenografted tumors was significantly inhibited in the nude mice. Disturbance of the ratio of Bax/Bcl-2 was found in the tumor tissues after kurarinone treatment, which was in accordance with the *in vitro* results. Moreover, expressions of the cleaved caspase-3, caspase-9 in tumor sections were also increased by kurarinone-treated xenograft mice model.

In summary, all the experimental results suggest that kurarinone has potent activity against human non-small cell lung cancer (NSCLC) A549 cell line. The regulation of cell apoptosis may take effects through the activation of apoptosis signaling pathway with mitochondria. Furthermore, kurarinone repressed ER stress and the activity of AKT in A549 cells, which suggested that A549 cell apoptosis is possibly implicated in this inhibition, and kurarinone have the potential to be AKT inhibitor in targeted chemotherapeutic drugs development. In addition, kurarinone has anticancer and apoptosis-inducing effects *in vivo*, and it could visibly reduce volume and weight of subcutaneous tumor masses. Besides, mitochondrial pathway was also involved in kurarinone-induced apoptosis *in vivo*.

## Ethics Statement

All animal experimental procedures were reviewed and approved by the Animal Ethical Committee of the Institute of Health and Epidemic Prevention (Wuhan, China; the protocol number 2017-SCUEC-AEC-0040), and animal care was conducted in accordance with institutional guidelines.

## Author Contributions

YR and XY contributed to the conception of the study equally. JY and HC contributed significantly to analysis and manuscript preparation equally. QW, SD, MH, XM, PS, and JD performed the data analyses and wrote the manuscript equally. YH and YW helped perform the analysis with constructive discussions equally.

## Conflict of Interest Statement

The authors declare that the research was conducted in the absence of any commercial or financial relationships that could be construed as a potential conflict of interest.

## References

[B1] Abu-Surrah A. S. Kettunen M. (2006). Platinum group antitumor chemistry: design and development of new anticancer drugs complementary to cisplatin. *Curr. Med. Chem.* 13 1337–1357. 10.2174/092986706776872970 16712474

[B2] Addie M. Ballard P. Buttar D. Crafter C. Currie G. Davies B. R. (2013). Discovery of 4-amino-*N*-[(1*S*)-1-(4-chlorophenyl)-3-hydroxypropyl]-1-(*7H*-pyrrolo[2,3-*d*]pyrimidin-4-yl)piperidine-4-carboxamide (AZD5363), an orally bioavailable, potent inhibitor of Akt kinases. *J. Med. Chem.* 56 2059–2073. 10.1021/jm301762v 23394218

[B3] Canel C. Moraes R. M. Dayan F. E. Ferreira D. (2000). Podophyllotoxin. *Phytochemistry* 54 115–120. 10.1016/S0031-9422(00)00094-7 10872202

[B4] Da Rocha A. B. Lopes R. M. Schwartsmann G. (2001). Natural products in anticancer therapy. *Curr. Opin. Pharmacol.* 1 364–369. 10.1016/S1471-4892(01)00063-7 11710734

[B5] Hill M. M. Adrain C. Duriez P. J. Creagh E. M. Martin S. J. (2004). Analysis of the composition, assembly kinetics and activity of native Apaf - 1 apoptosomes. *EMBO J.* 23 2134–2145. 10.1038/sj.emboj.7600210 15103327 PMC424369

[B6] Kang H. N. Kim S. H. Yun M. R. Kim H. R. Lim S. M. Kim M. S. (2016). ER2, a novel human anti-EGFR monoclonal antibody inhibit tumor activity in non-small cell lung cancer models. *Lung Cancer* 95 57–64. 27040853 10.1016/j.lungcan.2016.02.013

[B7] Kang T. H. Jeong S. J. Ko W. G. Kim N. Y. Lee B. H. Inagaki M. (2000). Cytotoxic lavandulyl flavanones from *Sophora flavescens*. *J. Nat. Prod.* 63 680–681. 10.1021/np990567x 10843587

[B8] Li H. Hu J. Wu S. Wang L. Cao X. Zhang X. (2016). Auranofin-mediated inhibition of PI3K/AKT/mTOR axis and anticancer activity in non-small cell lung cancer cells. *Oncotarget* 7 3548–3558. 10.18632/oncotarget.6516 26657290 PMC4823126

[B9] Li X. J. Kong D. X. Zhang H. Y. (2010). Chemoinformatics approaches for traditional Chinese medicine research and case application in anticancer drug discovery. *Curr. Drug Discov. Technol.* 7 22–31. 10.2174/157016310791162749 20156137

[B10] Mo S. S. Xiong H. Shu G. W. Yang X. Z. Wang J. X. Zheng C. (2013). Phaseoloideside E, a novel natural triterpenoid saponin identified from *Entada phaseoloides*, induces apoptosis in Ec-109 esophageal cancer cells through reactive oxygen species generation. *J. Pharmacol. Sci.* 122 163–175. 10.1254/jphs.12193FP 23782641

[B11] Mohan V. Agarwal R. Singh R. P. (2016). A novel alkaloid, evodiamine causes nuclear localization of cytochrome-c and induces apoptosis independent of p53 in human lung cancer cells. *Biochem. Biophys. Res. Commun.* 477 1065–1071. 10.1021/np0005457 27402273

[B12] Nakagawa T. Yuan J. Y. (2000). Cross-talk between two cysteine protease families. Activation of caspase-12 by calpain in apoptosis. *J. Cell Biol.* 150 887–894. 10.1083/jcb.150.4.887 10953012 PMC2175271

[B13] Niyazi M. Maihoefer C. Krause M. Rödel C. Budach W. Belka C. (2011). Radiotherapy and “new” drugs-new side effects? *Radiat. Oncol.* 6:177. 10.1186/1748-717X-6-177 22188921 PMC3266653

[B14] Pore M. M. Hilttermann T. J. Kruyt F. A. (2013). Targeting apoptosis pathways in lung cancer. *Cancer Lett.* 332 359–368. 10.1016/j.canlet.2010.09.012 20974517

[B15] Rao R. V. Hermel E. Castro-Obregon S. del Rio G. Ellerby L. M. Ellerby H. M. (2001). Coupling endoplasmic reticulum stress to the cell death program mechanism of caspase activation. *J. Biol. Chem.* 276 33869–33874. 10.1074/jbc.M102225200 11448953

[B16] Reddy R. K. Mao C. Baumeister P. Austin R. C. Kaufman R. J. Lee A. S. (2003). Endoplasmic reticulum chaperone protein GRP78 protects cells from apoptosis induced by topoisomerase inhibitors. *J. Biol. Chem.* 278 20915–20924. 10.1074/jbc.M212328200 12665508

[B17] Sai C. M. Li D. H. Xue C. M. Wang K. B. Hu P. Pei Y. H. (2015). Two pairs of enantiomeric alkaloid dimers from *Macleaya cordata*. *Org. Lett.* 17 4102–4105. 10.1021/acs.orglett.5b02044 26259683

[B18] Shen T. Li W. Wang Y. Y. Zhong Q. Q. Wang S. Q. Wang X. (2014). Antiproliferative activities of *Garcinia bracteata* extract and its active ingredient, isobractatin, against human tumor cell lines. *Arch. Pharm. Res.* 37 412–420. 10.1007/s12272-013-0196-1 23812779

[B19] Sun M. Y. Zuo J. Duan J. F. Han J. Fan S. M. Zhang W. (2008). Antitumor activities of kushen flavonoids in vivo and in vitro. *Zhong Xi Yi Jie He Xue Bao* 6 51–59. 10.3736/jcim20080111 18184547

[B20] Tophkhane C. Yang S. Bales W. Archer L. Osunkoya A. Thor A. D. (2007). Bcl-2 overexpression sensitizes MCF-7 cells to genistein by multiple mechanisms. *Int. J. Oncol.* 31 867–874. 10.3892/ijo.31.4.867 17786319

[B21] Wang C. Yang J. Zhao P. Zhou Q. Mei Z. N. Yang G. Z. (2014). Chemical constituents from *Eucalyptus citriodora* Hook leaves and their glucose transporter 4 translocation activities. *Bioorg. Med. Chem. Lett.* 24 3096–3099. 10.1016/j.bmcl.2014.05.014 24894556

[B22] Yang L. Q. Wang B. Gan H. Fu S. T. Zhu X. X. Wu Z. N. (2012). Enhanced oral bioavailability and anti-tumour effect of paclitaxel by 20 (s)-ginsenoside Rg3 in vivo. *Biopharm. Drug Dispos.* 33 425–436. 10.1002/bdd.1806 22898996

[B23] Yang X. Z. Huang M. Cai J. Y. Lv D. Lv J. N. Zheng S. J. (2017). Chemical profiling of anti-hepatocellular carcinoma constituents from *Caragana tangutica* Maxim. by off-line semi-preparative HPLC-NMR. *Nat. Prod. Res.* 31 1150–1155. 10.1080/14786419.2016.1230118 27626111

[B24] Yang X. Z. Wang C. Yang J. Wan D. R. Lin Q. X. Yang G. Z. (2014). Antimicrobial sesquiterpenes from the Chinese medicinal plant, *Chloranthus angustifolius*. *Tetrahedron Lett.* 55 5632–5634. 10.1016/j.tetlet.2014.08.052 26068434

[B25] Zhao N. Tian K. T. Cheng K. G. Han T. Hu X. Li D. H. (2016). Antiproliferative activity and apoptosis inducing effects of nitric oxide donating derivatives of evodiamine. *Bioorg. Med. Chem.* 24 2971–2978. 10.1016/j.bmc.2016.05.001 27178387

