# Supplementary material for: Inhibitory Effect of Kurarinone on Growth of Human Non-small Cell Lung Cancer: An Experimental Study Both in Vitro and in Vivo Studies
Source: Front Pharmacol. 2018 Mar 23;9:252. doi: 10.3389/fphar.2018.00252 (PMC5876310; doi:10.3389/fphar.2018.00252)

Inhibitory effect of kurarinone on growth of human non-small cell lung cancer: an experimental study both *in vitro* and *in vivo* studies

Jie Yang[1†], Hao Chen[1, 2†], Qiang Wang[1], Shihao Deng[1], Mi Huang[1], Xinhua Ma[1], Ping Song[3], Jingwen Du[1], Yun Huang[1], Yanzhang Wen[1], Yongshen Ren[1,*], Xinzhou Yang[1,*]

[1]School of Pharmaceutical Sciences, South Central University for Nationalities, Wuhan 430074, China

[2]College of Pharmacy, Guangxi University of Chinese Medicine, Nanning 530001, China

[3]Division of Science & Technology, Qinghai University for Nationalities, Xining 810007, China

[*]Corresponding authors at: School of Pharmaceutical Sciences, South-Central University for Nationalities, 182 Min-Zu Road, Wuhan 430074, China. Tel.: +86 27 67841196; Fax: +86 27 67841196.

E-mail addresses: godreny@scuec.edu.cn (Y.S. Ren); xzyang@mail.scuec.edu.cn (X.Z. Yang).

[†]These authors have contributed equally to this work.

Supplementary data Contents:

S1. ^{1}H NMR spectrum of kurarinone in DMSO

S2. ^{13}C NMR spectrum of kurarinone in DMSO

S3. ESIMS of kurarinone in DMSO

S4. Original images of Western blotting (Figure 3)

S5. Original images of Western blotting (Figure 4)

S6. Western blotting of AKT

S1. ^{1}H NMR spectrum of kurarinone in DMSO

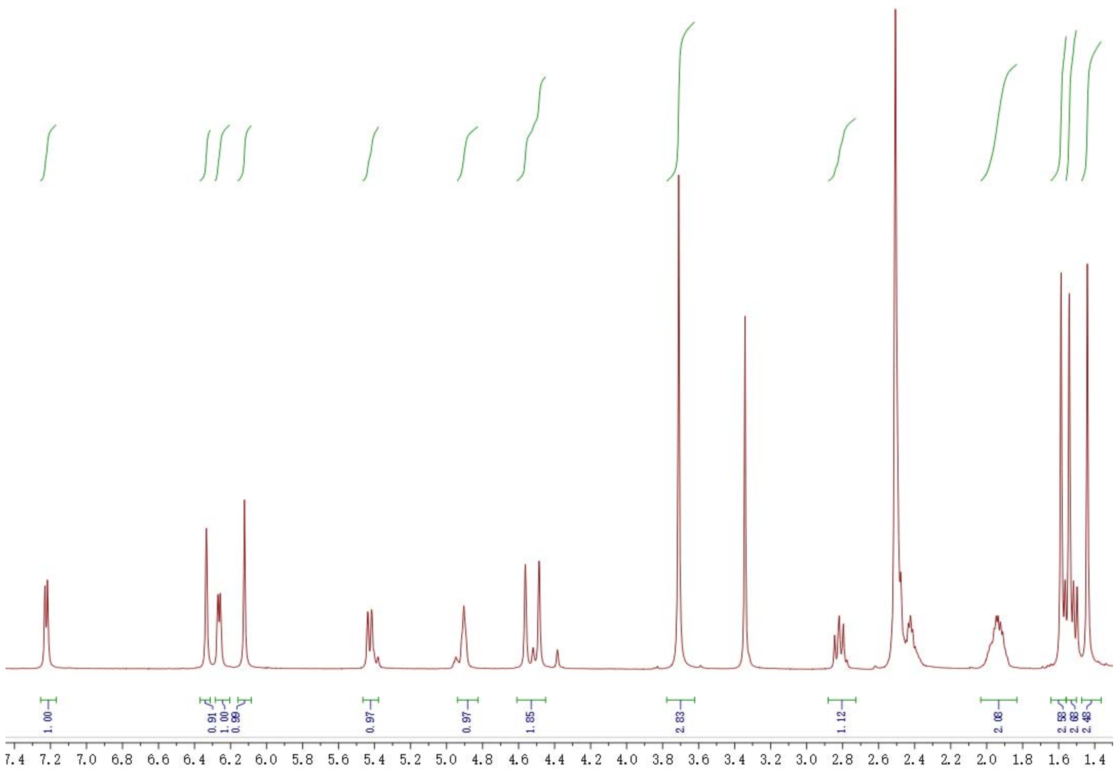

S2. ^{13}C NMR spectrum of kurarinone in DMSO

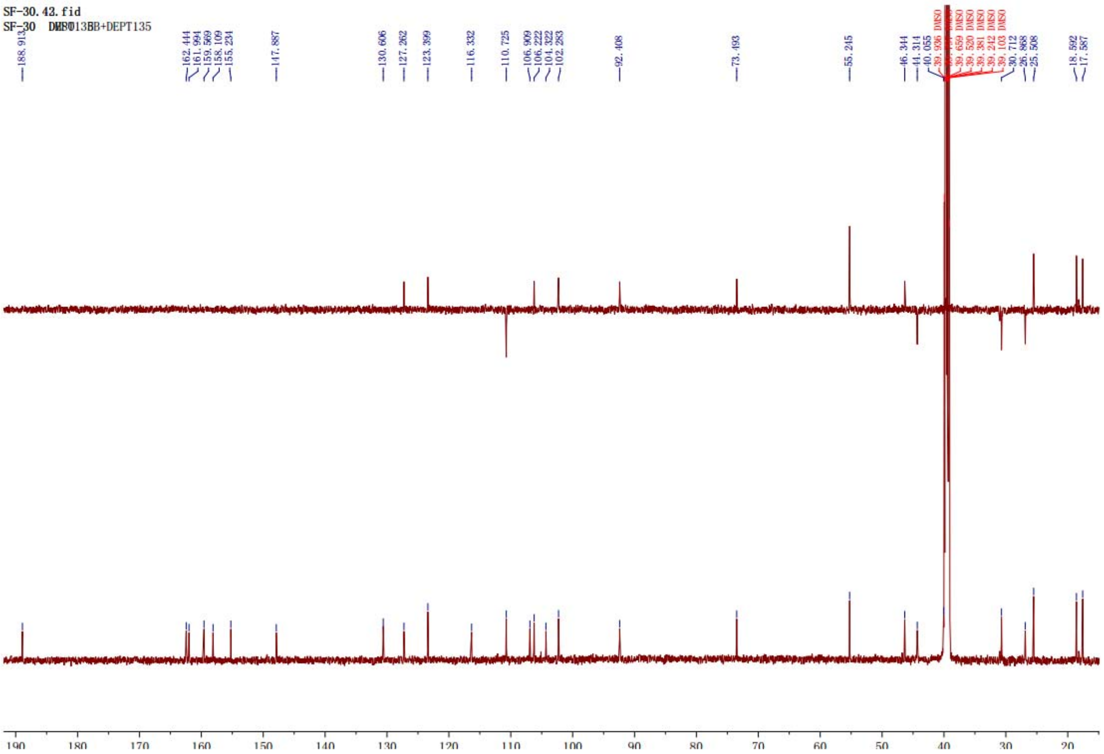

S3. ESIMS of kurarinone in DMSO

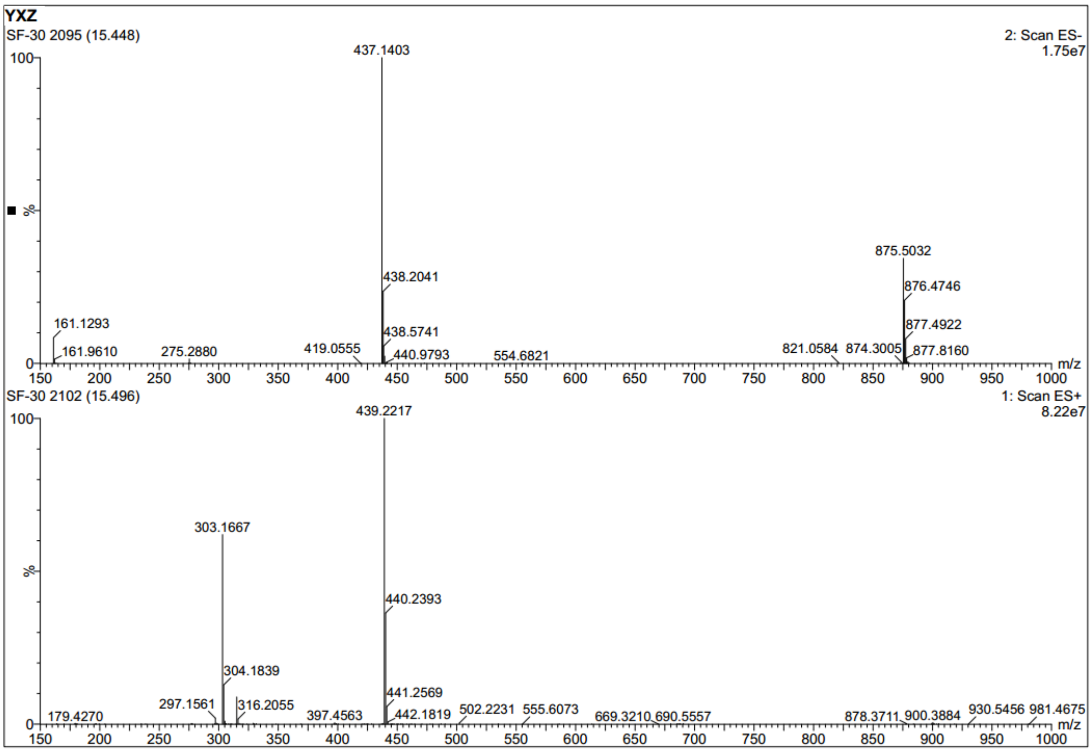

S4. Original images of Western blotting (Figure 3)

Figure 3

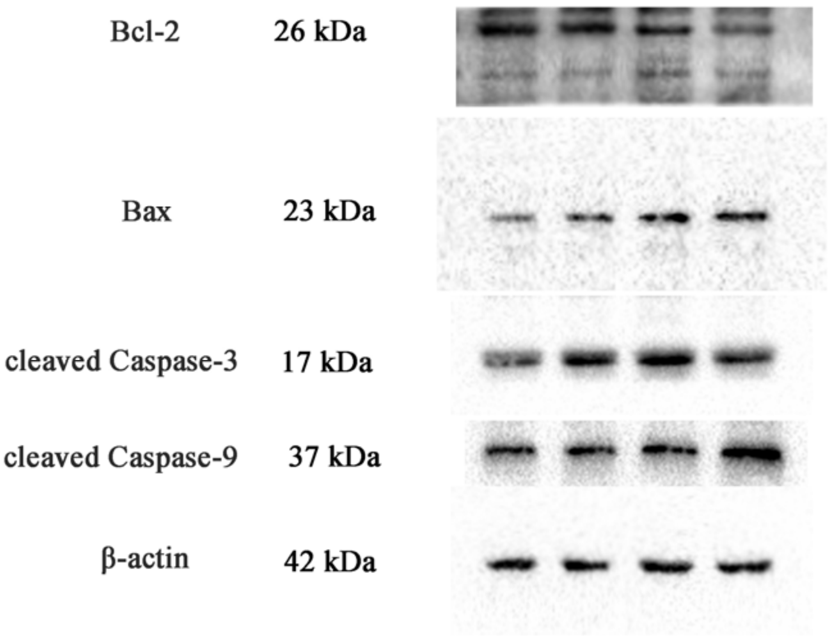

S5. Original images of Western blotting (Figure 4)

Figure 4

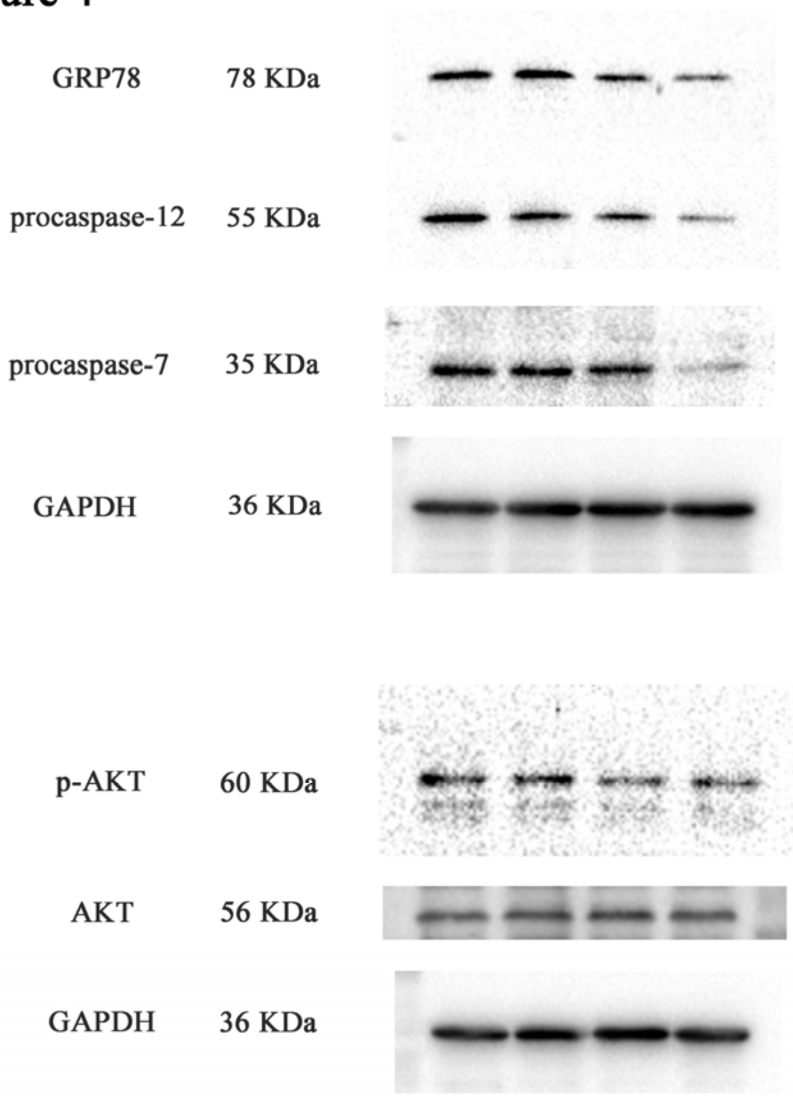

S6. Two repeated Akt western blot experiments

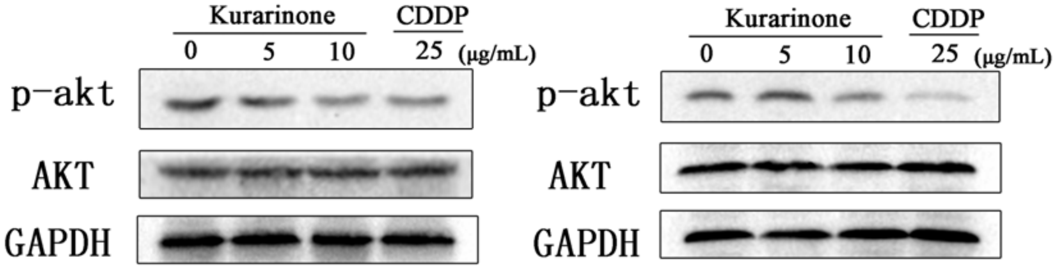

Supplement: Supplementary file 1 [file Presentation_1.PDF]
